# Pulmonary and Meningeal Cryptococcosis after Corticosteroid Therapy for Autoimmune Hepatitis: Coexistence of Cryptococci within Pulmonary Cancer Nodule

**DOI:** 10.1155/2013/807197

**Published:** 2013-07-10

**Authors:** Takashi Yuri, Ayako Kimura, Katsuhiko Yoshizawa, Yuko Emoto, Yuichi Kinoshita, Airo Tsubura

**Affiliations:** Department of Pathology II, Kansai Medical University, Hirakata, Osaka 573-1010, Japan

## Abstract

A case of autoimmune hepatitis complicated with pulmonary and meningeal cryptococcosis during long-term treatment with corticosteroid is reported. An 84-year-old woman who received long-term corticosteroid therapy (40 mg/day prednisolone for two years) for autoimmune hepatitis developed a headache, slight fever, and anorexia and was diagnosed with cryptococcal meningitis two months prior to hospital admission. Due to deterioration of her condition, the patient was transferred to our university hospital. After admission, a pulmonary nodule 1 cm in diameter was noticed in the patient's right lower lobe. Cryptococcal meningitis was diagnosed as positive for cryptococcal antigen from both serum and cerebrospinal fluid (CSF) as well as the growth of *Cryptococcus neoformans* (*C. neoformans*) in fungal culture. A combination therapy of amphotericin B and flucytosine was started, and the corticosteroid therapy was gradually reduced and finally discontinued. In addition to continuous cryptococcal infection, complications of *Pseudomonas aeruginosa* and methicillin-resistance *Staphylococcus aureus* infection caused death after a 2-month hospitalization. Autopsy disclosed encapsulated yeast in the lungs and subarachnoid space characteristic of *Cryptococcus*. The pulmonary nodule was found to be squamous cell carcinoma coexisting with *C. neoformans* within and around the cancer cell nests.

## 1. Introduction

*Cryptococcus* is a fungus found in most regions of the world, particularly in soil contaminated by bird droppings. Cryptococcal infection is usually acquired from the environment through the inhalation of contaminated particles into the lungs, which is followed by meningeal and cerebral involvement via a hematogeneous route. Cryptococcosis is usually seen in immunocompromised hosts such as HIV/AIDS patients, and is also seen in persons either with an intact immune system who have impaired cell-mediated immunity due to various chronic diseases or who receive high doses of corticosteroids for a long time. Patients with autoimmune disease who receive long-term corticosteroid therapy develop impaired cell-mediated immunity. Here, we describe a case of autoimmune hepatitis (AIH) complicated with pulmonary and meningeal cryptococcosis during treatment with corticosteroids. When a pulmonary nodule is seen in the lung, it is often difficult to distinguish pulmonary cryptococcosis from pulmonary neoplasia [1]. In the present case, a solitary pulmonary nodule was found during the course of treatment; *Cryptococcus neoformans (*C. neoformans*) * was found within and around cancer cell nests of squamous cell carcinoma.

## 2. Case Report

An 84-year-old woman who experienced appetite loss and general fatigue was found to have impaired liver function. Together with the presence of antinuclear antibody (ANA), she was diagnosed with AIH. Corticosteroid therapy (40 mg/day of prednisolone) improved her liver function. However, after two years of corticosteroid therapy, the patient developed cryptococcal meningitis, characterized by a 2-month history of headache, slight fever, and anorexia. Due to deterioration of her condition together with dysuria and dysphagia, the patient was transferred to Kansai Medical University Takii Hospital. She was admitted to our hospital due to a mild disturbance of consciousness, although brain infarction was deniable. After admission to our hospital, a pulmonary nodule 1 cm in diameter was found in the right lower lobe. The diagnosis of cryptococcal infection was confirmed by the presence of cryptococcal antigen (latex agglutination test) in the serum and cerebrospinal fluid (CSF) as well as the growth of *C. neoformans* in fungal culture. Prednisolone, which had been prescribed at a dosage of 40 mg/day for two years, was gradually reduced and finally discontinued, and a combination therapy of intravenous amphotericin B (6 mg/kg/day) and oral flucytosine (25 mg/kg × 4/day) was started. During the course of treatment, serum cryptococcal antigen titers were continuously positive. Aspiration pneumonia caused a general deterioration in the patient's condition. Accompanied by complications of *Pseudomonas aeruginosa* and methicillin-resistant *Staphylococcus aureus* infection, the patient died of septicemia after a 2-month hospitalization.

## 3. Autopsy Findings

**Figure 1:**
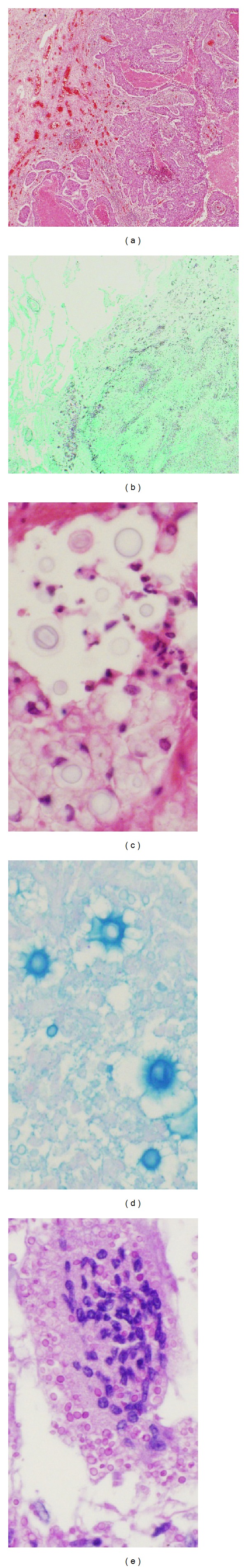
Pulmonary cryptococcosis. (a) Pulmonary nodule 1.2 × 1.0 cm in diameter is composed of keratinizing squamous cell carcinoma with necrosis (HE, ×20). (b) Yeast-like fungal bodies are scattered within and around the nests of squamous cell carcinoma cells (Grocott, ×20). (c) Well-developed *Cryptococcus neoformans* is seen within the area of tumor necrosis (HE, ×400). (d) Thick mucinous capsule is seen around the fungal body (same area as 1c; Alcian blue, ×400). (e) Multinucleated histiocytic cells with phagocytosed yeasts are haphazardly scattered within and around the nests of squamous cell carcinoma cells (PAS, ×400).

The pulmonary nodule that had been discovered in the right lower lobe after admission was grayish white in color and 1.2 × 1.0 cm in size and confirmed to be keratinizing squamous cell carcinoma (Figure 1(a)). Within and around the area of tumor necrosis and around the cancer cell nests, multiple nonmycelial yeast-like fungal bodies indicating the presence of cryptococci were easily identified by Grocott staining (Figure 1(b)). Well-developed, large *C. neoformans* with halo was identified in the necrotic area by hematoxylin and eosin staining (Figure 1(c)), periodic acid-Schiff staining, and Alcian blue staining (Figure 1(d)). Fungal bodies found adjacent to the cancer cell nests were either scattered freely in the stroma or phagocytosed in multinucleated histiocytes (Figure 1(e)); adjacent stroma showed a moderate amount of lymphocyte infiltration. However, *Cryptococcus* was hardly seen in other parts of the lung. Both non-cancerous parts of the lungs were compressed due to pleural effusions (500 mL each) with bronchopneumonia due to the aspiration of foreign substances into the bronchial tree. The regional lymph nodes were free of metastasis. In the central nervous system (CNS), *Cryptococcus* was present in subarachnoidal space but not in the brain parenchyma.

## 4. Discussion

Cryptococcosis is often seen in immunocompromised patients, but it also occurs in persons with intact immune systems who have an underlying chronic illness that has caused impaired cell-mediated immunity or who have been prescribed immunosuppressive drugs. In the present case, a patient who had been diagnosed as having AIH was treated with corticosteroid therapy for two years and had improved liver function and reduced symptoms but developed pulmonary and meningeal cryptococcosis. Long-term steroid therapy for AIH results in cryptococcal meningitis in 2% of patients (1/42 frequency) [5]. The inhalation of *Cryptococcus neoformans* causes primary pulmonary cryptococcosis followed by dissemination to the CNS via the hematogeneous route. *Cryptococcus* is the most common fungal infection of the CNS [6]. Cryptococcal meningitis patients with intact immune systems usually present with typical signs and symptoms of meningitis: fever, stiff neck, and headache [7]. A definitive diagnosis of cryptococcosis requires cryptococcal antigen detection, positive culture detection, and/or specific histopathological examination [8]. The diagnosis of *Cryptococcus* during the patient's lifetime is often difficult [9]. However, in the present case, a definitive diagnosis was made during the patient's lifetime by the presence of cryptococcal antigen (latex agglutination test) from serum and CSF and the growth of *C. neoformans* in fungal culture.

In the pulmonary nodule located in the right lower lobe, cryptococci leeching adjacent to and within the cancer cell nests were seen. Numerous cryptococci were haphazardly seen in the necrotic area around and within the cancer cell nests without any particular localization predilection; cryptococci were found throughout the stroma or were phagocytosed in the multinucleated histiocytes. Pulmonary cryptococcosis coexisting with pulmonary carcinoma is rare. Among 14 cases collected from the literature, the coexistence of *Cryptococcus* and carcinoma in the same lesion occurred in only four cases [1]. In some cases, *Cryptococcus* and cancer consist of different nodules, and in other cases, *Cryptococcus* and cancer coexist in the same nodule [1, 10]. Adding our case to Harada's review [1], the five cases are summarized in Table 1. All cases occurred in patients who were 71 years old or older, and the patients included both men and women. In most cases, the type of cancer was pulmonary adenocarcinomas, probably because the *Cryptococcus* may prefer residing in the peripheral lung, where adenocarcinomas frequently occur. Therefore, when a pulmonary nodule exists, the nodule may be a cryptococcoma, a neoplasia, or a combination in which *Cryptococcus* and cancer coexist in the same nodule.

*Cryptococcus* can be found in multiple organs (brain and spinal cord, lungs, lymph nodes, spleen, bone marrow, liver, kidneys, and adrenal glands) by autopsy, but the lungs and CNS are the organs most often involved [9]. Among CNS fungal infections, 116 of 129 cases (90%) are *Cryptococcus* meningitis [6]. CNS *Cryptococcus* has two forms, meningeal and parenchyma l [11]. However, organisms are present mostly in the subarachnoid space [9]. Meningitis is often the primary manifestation of CNS *Cryptococcus*, as in our case. Cryptococcal meningitis causes significant morbidity and mortality [12]. Comorbidities that could represent risk factors for cryptococcal meningitis include immunity disorders; the main debilitating conditions are HIV/AIDS, organ transplantations [13], hematological malignancies, anticancer chemotherapy, and chronic illnesses such as diabetes mellitus, systemic autoimmune disorders, chronic renal failure, sarcoidosis, alcoholism, and long-term steroid treatment [9, 14, 15]. For example, a 69-year-old woman developed cryptococcal meningoencephalitis after taking prednisolone (10 mg/day) for nine years for rheumatoid arthritis [14], and a 13-year-old girl developed cryptococcal meningitis after taking prednisolone (4.5 mg/kg body weight/day) for a year for autoimmune hemolytic anemia [15]. However, despite adequate treatment, cryptococcal antigen often remains positive at the one-year followup, indicating the necessity for prolonged treatment with antifungal drugs. In the present study, a patient who received 40 mg/day prednisolone for two years developed pulmonary and meningeal cryptococcosis. Long-term steroid therapy for AIH results in cryptococcal meningitis in 2% of patients (1/42 frequency).

Guidelines from the Infectious Diseases Society of America for the management of cryptococcal disease suggest the induction of consolidation therapy with amphotericin B followed by fluconazole [16]. Fluconazole is an adequate therapy for most patients who have infections that are limited to the lung [17]. Combination therapy with intravenous amphotericin B (due to poor penetration to CSF) and oral flucytosine (5-fluorocytosine), which penetrates the blood-brain barrier, is the treatment of choice for cryptococcal meningitis [6]. However, in the present case, intravenous amphotericin B and oral flucytosine could not adequately control the cryptococcosis, and the patient died of septicemia. *Cryptococcus* may affect patients with a wide spectrum of chronic diseases or long-term corticosteroid users whose immune systems are impaired. Therefore, the possibility of *Cryptococcus* should be considered with severely ill patients with immune impairment.

## Figures and Tables

**Table 1:** Coexistence of pulmonary cryptococcosis with pulmonary carcinoma within the same nodule.

Case	Age/sex	Location of tumor	Size (mm)	Histology of carcinoma	Reference
1	74/F	R-S6	95 × 65	ADC	[2]
2	74/M	L-LL	40 × 40 × 25	ADC	[3]
3	77/F	L-S8,9	ND	ADC	[4]
4	71/M	R-S2	14	ADC	[1]
5	84/F	R-LL	12 × 10	SQCC	Present case

R: right; L: left; S: segment; LL: lower lobe; ND: not described; ADC: adenocarcinoma; SQCC: squamous cell carcinoma.
